# Radiologic Evaluation of Oral Health Status in Patients Admitted to the Intensive Care Unit: A Multi-Institutional Retrospective Study

**DOI:** 10.3390/jcm13133913

**Published:** 2024-07-03

**Authors:** Yesel Kim

**Affiliations:** Department of Dental Hygiene, Jeonju Kijeon College, Jeonju 54989, Republic of Korea; yesel0912@gmail.com

**Keywords:** computed tomography, hospital dentistry, intensive care unit, oral care, oral hygiene

## Abstract

**Introduction:** Surveys distributed among intensive care unit (ICU) nurses reveal a significant need for dental care, with many acknowledging poor oral hygiene management. Poor oral health in ICU patients is linked to systemic problems, including aspiration pneumonia, necessitating pre-intervention assessments for bacterial diseases and dental risks. This study aims to evaluate the oral health status of ICU patients across three institutions through retrospective analysis. **Methods:** This retrospective study assessed the oral health status of ICU patients, using computed tomography (CT) images from three institutions over ten years. Through CT images, the oral status was evaluated in terms of total and lost tooth count and the presence of oral lesions (periapical lesions, cysts and tumors, caries, tartar, moderate to severe periodontal bone loss, tooth fractures). Variables included gender, age, the duration of ICU stay, and types of ICU. Statistical analysis was performed using chi-square tests, independent-sample *t*-tests, and logistic regression analysis. **Results:** Of the 450 participants, 430 were analyzed, revealing a prevalence of oral lesions in 67.0% of subjects. The prevalence of oral lesions was higher in males (71.5%) than females (57.7%, *p* = 0.006), and higher in those aged 40 and above (72.1%) compared to those under the age of 40 (47.8%, *p* < 0.001). This study found significant differences in oral health status based on gender, age, and ICU type, with surgical ICU patients generally having better oral health. Risk factors for oral lesions included gender, age, and duration of ICU stay. **Conclusions:** Most ICU patients have at least one oral lesion, regardless of the reason for their ICU admission. In particular, male ICU patients aged 40 and above have a higher prevalence of oral lesions, necessitating careful oral health assessment and treatment.

## 1. Introduction

Patients in intensive care unit (ICUs) often experience oral dryness due to medication, oral lesions, mastication and swallowing disorders, and the lack of voluntary oral hygiene, with many finding it difficult to communicate their needs [1,2]. According to a survey by RJG de Araujo among ICU nurses, 86% felt the need for dental care, and 98% agreed that a dental team should be present in the ICU [3]. Survey results among ICU nurses indicate that 53–58% admit to the poor management of patients’ oral hygiene, and about 80% neglect oral care compared to other body parts. Oral dental issues can significantly contribute to systemic problems by serving as reservoirs for pathogens [4,5]. Before interventions begin, it is crucial to eliminate bacterial diseases, especially since poor oral hygiene in ICU patients is closely linked to aspiration pneumonia [6,7,8,9]. It is noted that tooth loss, prosthesis dislodgement, tooth damage, and temporomandibular joint dislocation often occur in patients with intubation, endoscopy, or oral devices for airway maintenance, yet these risks are rarely assessed beforehand [4].

Many inpatients in the ICU present with side effects and various conditions, including hyposalivation associated with medications, chewing and swallowing discomfort due to antineoplastic treatments, the presence of oral lesions caused by systemic diseases, and difficulty in maintaining oral hygiene during hospitalization [1,2,3]. Unfortunately, oral hygiene kits are insufficient and nursing teams are not qualified or trained to perform oral hygiene and provide proper oral care instructions [1]. It is not well known when a dentist should intervene in the hospital environment [3]. ICU patients find it challenging to visit the dentist, and poor oral hygiene is very common in ICU settings. A systematic review and meta-analysis in 2011 reported that the plaque index of hospitalized patients significantly increased, rising from 23% upon admission to 93% after 10 days of hospitalization [4]. Additionally, two studies reported a severe increase in the gingival inflammation index [5,6]. Particularly in the ICU, where monitoring organs and systems to prevent deterioration is crucial [3], the condition of the oral cavity is also important, emphasizing the role of hospital dentistry. Dentists and dental hygienists also work in multidisciplinary teams, especially in the hospital, which is essential when the ultimate goal is improving the quality of life of patients [7].

The 2020 Korean Nurses Association guidelines for oral care aim to enable patients to manage their own oral hygiene [8]. However, from a dental perspective, self-managed oral hygiene does not guarantee cleanliness. The guidelines recommend dental care in cases where patients do not express symptoms (pain, tooth mobility, and bleeding during brushing), making it challenging for nurses without dental expertise to perform accurate assessments (gingival swelling, calculus, discoloration of teeth or tongue, and ill-fitting prostheses) [9]. International studies show that 82.5% of patients admitted for non-dental reasons require active dental treatment, and 58.6% need invasive dental interventions, with over 62.2% at risk of oral complications during their stay and more than 34% harboring acute conditions (abscess drainage, tooth mobility, toothache, and the need for extraction) [9]. Nursing teams’ criteria for assessing oral conditions traditionally follow the Oral Assessment Guide (OAG) proposed by Eilers [10]. This assessment guideline is based on nurses’ observations using sight, hearing, and touch, but it excludes the evaluation of periodontal disease and dental caries, which are the most common bacterial diseases in the oral cavity. Therefore, it is necessary to assess the oral condition of ICU patients from a dental perspective and determine if more proactive dental intervention is needed beyond the current level of oral hygiene management based on the assessment results. The purpose of this study is to evaluate the oral health status of patients admitted to the ICUs of three domestic institutions over the past ten years through a multi-institutional retrospective analysis.

## 2. Material and Methods

This study was approved by the Institutional Review Board at Armed Forces Capital Hospital (AFCH-21-IRB-009), Seoul National University Bundang Hospital (B-2106/691-105), Gangnam Severence Hospital (3-2021-0199). This study was conducted according to the principles of the Declaration of Helsinki for research on humans.

The oral health status of patients admitted to the ICU from 1 March 2011 to 31 March 2021 was retrospectively assessed using facial CT, neck CT, and mandible CT images taken either during their ICU stay or immediately before ICU admission. The selection criteria were as follows: (1) individuals with at least one recorded image of facial CT, neck CT, or mandible CT, taken during their stay in the emergency room or while admitted to the ICU; and (2) adults aged 19 or older with a history of ICU admission. Exclusion criteria included (1) patients under 19, (2) patients with congenital deformities in the maxillofacial area, (3) patients admitted to the ICU under a dental primary care provider, and (4) patients without radiographic images available for evaluating oral status. A total of 450 individuals were selected for this multi-institutional study across three research institutions, with 20 individuals excluded due to unreadable images, resulting in a final sample size of 430 for analysis. 

**Figure 1:**
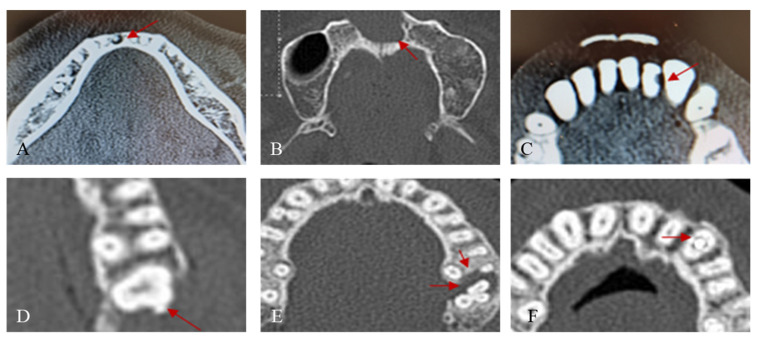
Representative oral lesion criteria in CT image. (**A**) Periapical abscess. (**B**) Cystic lesion. (**C**) Dental caries. (**D**) Calculus. (**E**) Periodontitis (**F**) Tooth fracture.

Oral lesions were identified by screening cross-sectional and longitudinal sections of the maxilla and mandible based on the oral plane in head and neck CT (facial CT, neck CT, and mandible CT), evaluating the number of remaining and lost teeth, and assessing the presence or absence of six types of oral lesions as per the defined criteria (Figure 1). The outcomes included (1) total and lost tooth counts of the subjects; (2) average MT index (missing permanent teeth index) per subject in the study group; and (3) the presence of oral lesions (periapical lesions, cysts and tumors, caries, calculus, moderate to severe periodontal bone loss, and tooth fractures). The variables included (1) gender, (2) age, (3) duration of ICU stay, (4) primary department at admission, and (5) type of ICU (surgical/medical ICU). The imaging exmainations were conducted by a dentist (J.K.Ku)

Descriptive statistics and frequency analyses were conducted for general characteristics. Differences in the number of remaining and lost teeth, assessed according to general characteristics, were analyzed using chi-square tests and independent-sample *t*-tests. Logistic regression analysis was performed to identify risk factors for the presence of oral lesions. Data are expressed as means ± standard deviations and analyzed using IBM SPSS Statistics 25.0 (IBM, Armonk, NY, USA).

## 3. Results

Out of the 450 study participants, 20 were excluded due to non-diagnostic images, leaving 430 (58 patients in Armed Forces Capital Hospital, 238 patients in Seoul National University Bundang Hospital, and 150 patients in Gangnam Severence Hospital) patients for final analysis. The head and neck CTs used for analysis comprised 48.1% neck CTs and 51.9% facial CTs. The gender distribution was 67% male and 33% female, with an average age of 56.6 years. The average ICU stay was 18.6 days, with surgical ICU types accounting for 83.7% of cases. In the CT scans of all patients, oral lesions were found in 67.0% of cases (Figure 1). The frequency of the discovered oral lesions was as follows: periapical abscess (41.4%), periodontitis with bone defect (37.4%), dental caries (22.8%), tooth fracture (14.9%), calculus (6.0%), and cystic lesion (2.8%). The results are given in descending order.

### 3.1. Differences in General Characteristics by Gender

The results of assessing the different types of medical department by gender showed that both men and women were most frequently seen in surgical departments. The type of ICU showed that the emergency department frequency was the highest for both men and women, with statistically significant differences (Table 1).

### 3.2. Differences in General Characteristics by Age

When assessing differences in institutions by age, the results showed statistically significant differences. The type of medical department visited for all three age groups was most frequently surgical (Table 2).

### 3.3. Evaluation of Oral Condition through the Analysis of Medical Records

#### 3.3.1. Evaluation of Oral Condition of the Subjects through the Analysis of Medical Records

The average number of remaining teeth in the subjects was 23.2, with an average of 4.8 teeth lost (Table 3). The presence of oral lesions, as determined through CT image analysis, showed that 33% of subjects had at least one lesion.

#### 3.3.2. Differences in Oral Health Status Assessment According to General Characteristics

When applied among study participants, the results of the Missing Permanent Teeth Index (MPTI) varied according to the general characteristics, with the highest MPTI observed at Bundang Seoul National University Hospital at 5.6, and the lowest at Gangnam Severance Hospital at 3.7. The MPTI increased with age, showing more than 8 teeth missing in individuals aged 60 and over compared to those aged 19–39. The index score was higher in the medical department, standing at 7.2, compared to the surgical department (Table 4).

The assessment of oral health status by gender showed that men had a higher frequency of having at least one oral lesion compared to women, and men also showed a higher frequency of tooth fractures (Table 5). Age-related assessments of oral health status revealed significant differences in the number of remaining and missing teeth. Furthermore, the presence of oral lesions increased with age, showing a statistically significant difference in terms of the frequency of having at least one lesion (Table 6). The assessment of oral health status by ICU type showed that the surgical department had a higher average number of remaining teeth (23.7) compared to the medical department (20.8), with statistically significant differences. Tooth fractures among oral lesions were more frequent in the surgical department, showing a statistically significant difference (Table 7).

#### 3.3.3. Risk Factors Influencing the Prevalence of Oral Lesions

To identify the risk factors affecting the prevalence of oral lesions, logistic regression analysis was performed, focusing on variables that showed significance among oral examinations and general characteristics. The regression model was statistically significant (Hosmer and Lemeshow x^2^ = 7.189, *p* = 0.515), with an explanatory power of approximately 12.6% (Nagelkerke R^2^ = 0.126). The significance test of regression coefficients revealed that gender, age, and the duration of ICU stay have a significant impact on the occurrence of oral lesions. The prevalence of oral lesions increases by 1.03 times with each increase in age (Table 8).

## 4. Discussion

This study investigates the oral health of ICU patients over ten years across three institutions, recognizing that such patients often experience oral health issues due to factors like medication effects, lack of voluntary hygiene, and medical interventions. Oral health status reveals significant oral health issues among ICU patients, with the average number of remaining teeth being 23.2 and an average loss of 4.8 teeth. Sixty-seven percent of subjects had at least one oral lesion, detected via CT. The missing permanent teeth (MT) index score varied by age, hospital days, and types of ICU, with higher indices observed in older patients and those undergoing medical care. Oral lesions increased with age, and males were more likely to have them, including tooth fractures. Notably, patients aged 40 and older showed a significantly higher prevalence of dental abscesses, caries, and advanced periodontitis, highlighting the urgent need for proactive dental treatments and policy development for oral evaluation and management in ICU settings.

In 2021, Jun et al. published a review of studies on the oral health status of ICU patients [11]. Their systematic review identified indicators used to assess oral health from a dental perspective, including the plaque index [4,12], periodontitis (probing depth, bleeding on probing) [12,13], the number of teeth lost [12], dental caries [13], tooth fracture [13], odontogenic abscess [13], gingivitis [13], oral candidiasis [13,14], and mucositis [13], along with evaluations of the need for dental intervention [14,15,16]. These studies found that 65–90% of cases required dental treatment, diagnosed through visual and tactile examination by dental professionals. Bellissimo-Rodrigues et al. reported that gingivitis was the most frequently occurring oral condition requiring treatment [13]. Given that gingivitis, a bacterial disease, can increase the risk of ventilator-associated pneumonia (VAP) if left untreated due to plaque accumulation, The Center for Disease Control and Prevention recommends managing oral hygiene [17]. However, varieties of periodontitis with bone destruction, periapical abscess, cracked tooth, and tooth fracture are more serious dental diseases than gingivitis, and they cannot be resolved with oral hygiene management alone, requiring professional treatment by a dentist.

**Figure 2:**
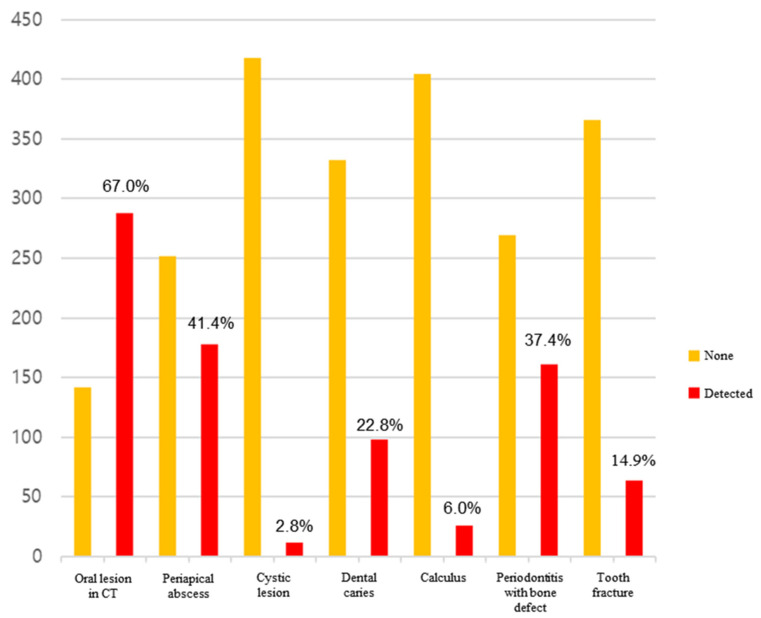
Frequency of oral lesions found via CT.

This study was conducted via examinations, using CT scans to determine the extent to which patients admitted to the ICU were affected by these serious conditions. Oral lesions requiring treatment were found in 66.97% (288/430) of patients, with an average of 1.5 oral lesions per patient (Figure 2). Among them, the three most frequently occurring conditions, periapical abscess (41.4%), periodontitis with bone defect (37.4%), and dental caries (22.8%), can cause severe symptoms if not treated aggressively. Additionally, they are more serious bacterial diseases than gingivitis. In particular, periapical abscesses can be difficult to diagnose without radiographic images, suggesting that a considerable number of ICU patients may be underdiagnosed. Additionally, many studies report that interventions by dental professionals significantly reduce the incidence of pneumonia and mortality due to respiratory diseases [13,17,18,19]. This study discovered that individuals aged 60 and above, particularly males (Table 8), face a statistically significant higher risk of developing oral lesions. Considering the frequency of gingivitis, candidiasis, mild calculus, and mild periodontitis that are not visible in radiographs, the need for active dental care among ICU patients is likely to increase. Consequently, it may be advisable to initially screen ICU patients in order to assess their oral health using radiographs or dental exams. This approach would enable immediate proactive dental treatment upon admission, especially for elderly male patients.

## 5. Conclusions

This study, based on a decade of record analysis, confirms that 67% of ICU patients had at least one oral lesion detectable on radiographs, with male patients aged 40 and older showing a significantly higher prevalence of oral lesions. This underscores the urgent need for proactive dental treatments and highlights the necessity of developing policies for oral evaluation and management in ICU patients.

## Figures and Tables

**Table 1:** Differences in general characteristics by gender.

	Male (n = 288)	Female (n = 142)	*p*
ICU admission duration			
1–6 days	93 (49.2%)	59 (55.1%)	0.058
7–13 days	33 (17.5%)	8 (7.5%)	
≥14 days	63 (33.3%)	40 (37.4%)	
Types of ICU			
Medical	37 (12.8%)	33 (23.2%)	0.009
Surgical	251 (87.2%)	109 (76.8%)	
Subtypes of ICU			
Cardiac	13 (4.5%)	6 (4.2%)	<0.001
Emergency	79 (27.4%)	51 (35.9%)	
Medical	23 (8.0%)	30 (21.1%)	
Neurological	69 (24.0%)	30 (21.1%)	
Surgical	66 (22.9%)	18 (12.7%)	
Trauma	38 (13.2%)	7 (4.9%)	

Assessed by chi-square test.

**Table 2:** Differences in general characteristics by age.

	19–39 Years (n = 92)	40–59 Years (n = 144)	>60 Years (n = 193)	*p*
Gender				
Male	71 (77.2%)	95 (66.0%)	121 (62.7%)	0.050
Female	21 (22.8%)	49 (34.0%)	72 (37.3%)	
ICU admission duration				
1–6 days	32 (48.5%)	45 (54.9%)	74 (50.3%)	0.432
7–13 days	12 (18.2%)	13 (15.9%)	16 (10.9%)	
≥14 days	22 (33.3%)	24 (29.3%)	57 (38.8%)	
Types of ICU				
Medical	6 (6.5%)	18 (12.5%)	46 (23.8%)	<0.001
Surgical	86 (93.5%)	126 (87.5%)	147 (76.2%)	
Subtypes of ICU				
Cardiac	1 (1.1%)	6 (4.2%)	12 (6.2%)	<0.001
Emergency	16 (17.4%)	43 (29.9%)	71 (36.8%)	
Medical	4 (4.3%)	12 (8.3%)	37 (19.2%)	
Neurological	31 (33.7%)	32 (22.2%)	36 (18.7%)	
Surgical	26 (28.3%)	35 (24.3%)	22 (11.4%)	
Trauma	14 (15.2%)	16 (11.1%)	15 (7.8%)	

Assessed by chi-square test.

**Table 3:** Evaluation of oral condition of the subjects through analysis of medical records.

	Mean ± SD
Number of remained teeth	
Maxilla	11.4 ± 4.1
Mandible	11.8 ± 3.4
Total	23.2 ± 6.8
Number of missing teeth	
Maxilla	2.6 ± 4.1
Mandible	2.2 ± 3.4
Total	4.8 ± 6.8
MT index	4.80
Numbers of oral lesion per person	1.5 ± 1.8

**Table 4:** Index of missing teeth according to general characteristics.

	Numbers of Patient	Numbers of Missing Teeth	MT Index
Gender			
Male	288	1330	4.62
Female	142	733	5.16
ICU admission duration			
1–6 days	92	78	0.85
7–13 days	144	426	2.96
≥14 days	193	1559	8.08
Types of ICU			
Medical	70	504	7.20
Surgical	360	1559	4.33
Subtypes of ICU			
Cardiac	19	154	8.11
Emergency	130	705	5.42
Medical	53	381	7.19
Neurological	99	346	3.49
Surgical	84	264	3.14
Trauma	45	211	4.69

**Table 5:** Index of missing permanent teeth according to gender.

	Male (n = 288)	Female (n = 142)	*p*
Number of remained teeth			
Maxilla	11.5 ± 4.0	11.1 ± 4.2	0.313
Mandible	11.8 ± 3.3	11.7 ± 3.4	0.733
Total	23.4 ± 6.7	22.8 ± 7.0	0.437
Number of missing teeth			
Maxilla	2.5 ± 4.0	2.9 ± 4.2	0.313
Mandible	2.2 ± 3.3	2.3 ± 3.4	0.733
Total	4.6 ± 6.7	5.2 ± 7.0	0.437
Oral lesion in CT images			
None	82 (28.5%)	60 (42.3%)	0.006
Detected	206 (71.5%)	82 (57.7%)	
Periapical abscess			
None	158 (54.9%)	94 (66.2%)	0.032
Detected	130 (45.1%)	48 (33.8%)	
Cystic lesion			
None	281 (97.6%)	137 (96.5%)	0.738
Detected	7 (2.4%)	5 (3.5%)	
Dental caries			
None	217 (75.3%)	115 (81.0%)	0.235
Detected	71 (24.7%)	27 (19.0%)	
Calculus			
None	270 (93.8%)	134 (94.4%)	0.970
Detected	18 (6.2%)	8 (5.6%)	
Periodontitis (more than moderate alveolar bone destruction)			
None	175 (60.8%)	94 (66.2%)	0.323
Detected	113 (39.2%)	48 (33.8%)	
Tooth fracture			
None	237 (82.3%)	129 (90.8%)	0.028
Detected	51 (17.7%)	13 (9.2%)	
Numbers of oral lesion	1.6 ± 1.9	1.3 ± 1.8	0.116

Assessed by chi-square test.

**Table 6:** Index of missing permanent teeth according to age.

	19–39 Years (n = 92)	40–59 Years (n = 144)	≥60 Years (n = 193)	*p*
Number of remained teeth				
Maxilla	13.7 ± 1.0	12.5 ± 2.9	9.5 ± 4.9	<0.001
Mandible	13.5 ± 1.0	12.5 ± 2.2	10.4 ± 4.2	<0.001
Total	27.2 ± 1.7	25.0 ± 4.6	19.9 ± 8.1	<0.001
Number of missing teeth				
Maxilla	0.3 ± 1.0	1.5 ± 2.9	4.5 ± 4.9	<0.001
Mandible	0.5 ± 1.0	1.5 ± 2.2	3.6 ± 4.2	<0.001
Total	0.8 ± 1.7	3.0 ± 4.6	8.1 ± 8.1	<0.001
Oral lesion in CT images				
None	48 (52.2%)	39 (27.1%)	55 (28.5%)	<0.001
Detected	44 (47.8%)	105 (72.9%)	138 (71.5%)	
Periapical abscess				
None	66 (71.7%)	81 (56.2%)	105 (54.4%)	0.016
Detected	26 (28.3%)	63 (43.8%)	88 (45.6%)	
Cystic lesion				
None	90 (97.8%)	139 (96.5%)	188 (97.4%)	0.817
Detected	2 (2.2%)	5 (3.5%)	5 (2.6%)	
Dental caries				
None	79 (85.9%)	108 (75.0%)	145 (75.1%)	0.090
Detected	13 (14.1%)	36 (25.0%)	48 (24.9%)	
Calculus				
None	86 (93.5%)	136 (94.4%)	182 (94.3%)	0.948
Detected	6 (6.5%)	8 (5.6%)	11 (5.7%)	
Periodontitis (more than moderate alveolar bone destruction)				
None	72 (78.3%)	87 (60.4%)	110 (57.0%)	0.002
Detected	20 (21.7%)	57 (39.6%)	83 (43.0%)	
Tooth fracture				
None	77 (83.7%)	122 (84.7%)	167 (86.5%)	0.795
Detected	15 (16.3%)	22 (15.3%)	26 (13.5%)	
Numbers of oral lesion	0.9 ± 1.3	1.5 ± 1.4	1.8 ± 2.2	<0.001

Assessed by chi-square test.

**Table 7:** Oral status according to the types of ICU.

	Medical (n = 70)	Surgical (n = 360)	*p*
Number of remained teeth			
Maxilla	10.0 ± 5.1	11.7 ± 3.8	0.009
Mandible	10.8 ± 4.1	12.0 ± 3.2	0.027
Total	20.8 ± 8.5	23.7 ± 6.4	0.008
Number of missing teeth			
Maxilla	4.0 ± 5.1	2.3 ± 3.8	0.009
Mandible	3.2 ± 4.1	2.0 ± 3.2	0.027
Total	7.2 ± 8.5	4.3 ± 6.4	0.008
Oral lesion in CT images			
None	28 (40.0%)	114 (31.7%)	0.223
Detected	42 (60.0%)	246 (68.3%)	
Periapical abscess			
None	44 (62.9%)	208 (57.8%)	0.511
Detected	26 (37.1%)	152 (42.2%)	
Cystic lesion			
None	69 (98.6%)	349 (96.9%)	0.719
Detected	1 (1.4%)	11 (3.1%)	
Dental caries			
None	52 (74.3%)	280 (77.8%)	0.630
Detected	18 (25.7%)	80 (22.2%)	
Calculus			
None	67 (95.7%)	337 (93.6%)	0.688
Detected	3 (4.3%)	23 (6.4%)	
Periodontitis (more than moderate alveolar bone destruction)			
None	46 (65.7%)	223 (61.9%)	0.645
Detected	24 (34.3%)	137 (38.1%)	
Tooth fracture			
None	65 (92.9%)	301 (83.6%)	0.071
Detected	5 (7.1%)	59 (16.4%)	
Numbers of oral lesion	1.3 ± 1.4	1.6 ± 1.9	0.112

Assessed by chi-square test.

**Table 8:** Risk factors affecting the prevalence of oral lesions.

Variables (ref.: None)	B	SE	OR	95% CI	*p*
Gender	−0.753	0.229	0.471	(0.300~0.738)	0.001
Age	0.030	0.007	1.030	(1.016~1.044)	0.000
Types of ICU	0.513	0.295	1.671	(0.938~2.976)	0.081
ICU admission day	−0.009	0.004	0.991	(0.983~0.998)	0.013
Numbers of remained teeth	0.012	0.019	1.012	(0.975~1.052)	0.522
-2LL = 504.550, Nagelkerke R^2^ = 0.126, Hosmer and Lemeshow x^2^ = 7.198 (*p* = 0.515)					

OR: odds ratio, 95% CI: 95% confidence interval. Assessed by logistic regression analysis.

## Data Availability

Not applicable.
